# Incidentally discovered developmental venous anomaly with an associated white matter changes in a 7-year-old girl: a case report and literature review

**DOI:** 10.1093/jscr/rjag009

**Published:** 2026-01-28

**Authors:** Khulood K AlRaddadi, K K Alraddadi, Nouf Koshak, N Koshak, Wael Alshaya, W Alshaya

**Affiliations:** Pediatric Neurosurgery Division, Pediatric Surgery Department, King Abdullah Specialized Children's Hospital (KASCH), Ministry of National Guard Health Affairs, ArRimayah District, PO Box 22490, Riyadh 11426, Riyadh Province, Saudi Arabia; Pediatric Neurosurgery Division, Pediatric Surgery Department, King Abdullah Specialized Children's Hospital (KASCH), Ministry of National Guard Health Affairs, ArRimayah District, PO Box 22490, Riyadh 11426, Riyadh Province, Saudi Arabia; Neurosurgery Division, Surgery Department, King Saud University, PO Box 59220, Riyadh 11525, Saudi Arabia; Neurosurgery Division, Surgery Department, King Saud University, PO Box 59220, Riyadh 11525, Saudi Arabia; Pediatric Neurosurgery Division, Pediatric Surgery Department, King Abdullah Specialized Children's Hospital (KASCH), Ministry of National Guard Health Affairs, ArRimayah District, PO Box 22490, Riyadh 11426, Riyadh Province, Saudi Arabia; Pediatric Neurosurgery Division, Pediatric Surgery Department, King Abdullah Specialized Children's Hospital (KASCH), Ministry of National Guard Health Affairs, ArRimayah District, PO Box 22490, Riyadh 11426, Riyadh Province, Saudi Arabia

**Keywords:** developmental venous anomaly, vascular malformation, venous angioma, cavernous malformation, magnetic resonance imaging

## Abstract

Developmental venous anomalies (DVAs) are the most common cerebral vascular malformation, with age-dependent prevalence variation in the pediatric population. We report a case of an incidentally discovered left anterior temporal DVA with associated white matter changes in a previously healthy 7-year-old girl following minor head trauma. Initial computed tomography revealed a temporal lobe hypodensity, prompting magnetic resonance imaging (MRI), which demonstrated a classic DVA with adjacent T2/Fluid-Attenuated Inversion Recovery (FLAIR) hyperintense white matter changes without restricted diffusion, blooming susceptibility, or abnormal enhancement. The patient remained asymptomatic throughout follow-up, with serial magnetic resonance imaging showing stability of both the DVA and parenchymal abnormalities. The association of DVAs with white matter changes is attributed to chronic venous hypertension or altered hemodynamics. While generally benign, DVAs can occasionally cause seizures or rarely hemorrhage in children. This case highlights the characteristic imaging features and typically benign course of DVAs with white matter changes in children, supporting conservative management with clinical and radiological surveillance.

## Introduction

Developmental venous anomalies (DVAs), formerly known as venous angiomas, are the most common congenital cerebral vascular malformations. They consist of multiple medullary veins converging radially into a larger collecting vein that drains into the dural sinus or deep venous system, creating a characteristic ‘caput medusae’ appearance on contrast-enhanced imaging. DVAs arise due to arrested embryonic development of the cerebral venous system, with persistence of primitive medullary veins that would otherwise regress during brain maturation [1–4].

Magnetic resonance imaging (MRI) studies report that DVA prevalence ranges from 2.6% to 9.6%, showing a marked age-related increase, particularly in children. This suggests postnatal maturation and myelination influence the detectability or evolution of DVAs [5–8]. Most DVAs are solitary, though multiple lesions occur in about 7%–16% of cases, sometimes associated with genetic syndromes [7, 9].

Importantly, ~12.5% of DVAs are associated with adjacent white matter T2-weighted and FLAIR hyperintensities. These brain parenchymal signal changes likely result from chronic venous hypertension, altered hemodynamics, or relative ischemia in the drainage territory [10–13]. Clinically, DVAs are usually incidental findings and asymptomatic, with hemorrhage risk <1% per year. Occasionally, seizures or neurological deficits arise, particularly in pediatric patients [5, 14].

This report details the incidental discovery of a left temporal DVA with associated white matter changes in a 7-year-old girl, emphasizing pediatric cerebrovascular imaging findings.

## Case presentation

A 7-year-old girl with no significant past medical history presented after two minor head trauma events on the same day affecting her left temporal region. The first was a slip causing a direct impact, followed by a push from her sibling, after which she developed vomiting episodes. Physical and neurological examinations were normal. A non-contrast computed tomography (CT) scan excluded acute hemorrhage but revealed an ill-defined hypodense area in the left inferior temporal lobe.

**Figure 1:**
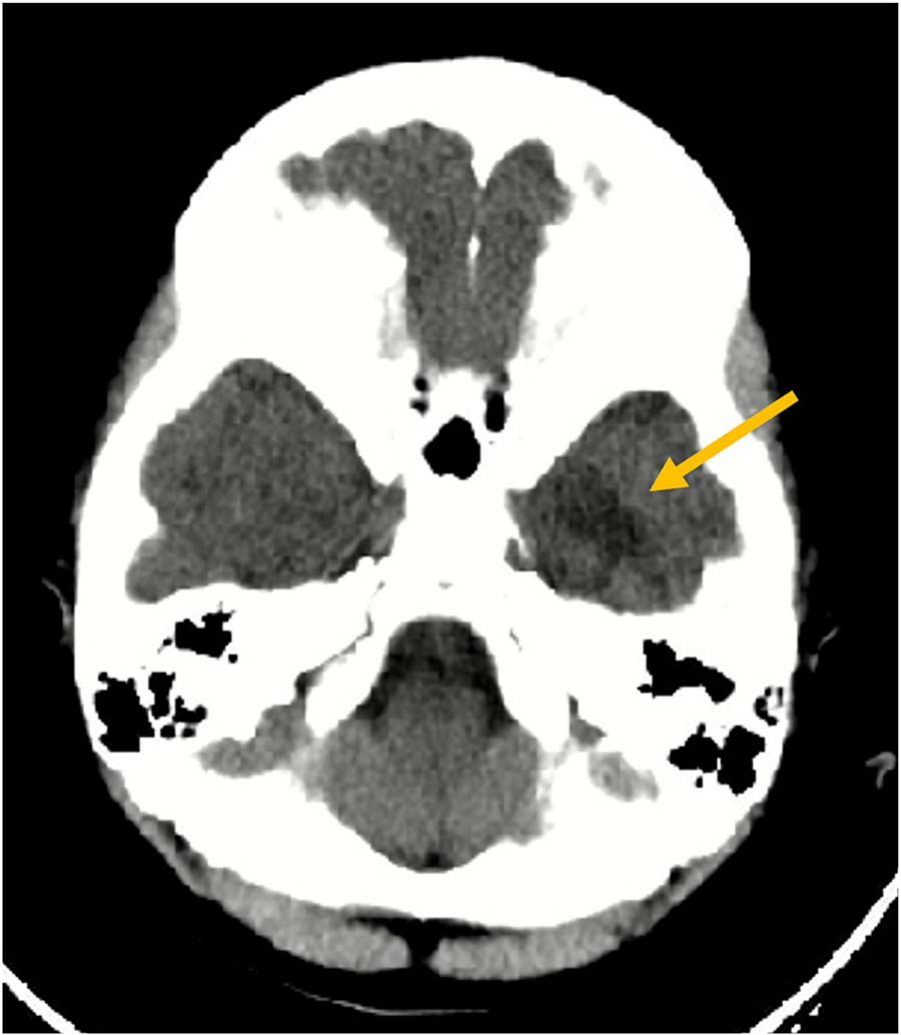
Axial non-contrast CT scan of the brain. This image demonstrates an ill-defined hypodensity (arrow) in the left inferior temporal lobe (seen as a darker area). No acute intracranial hemorrhage or fracture was identified at the time of this initial scan.

**Figure 2:**
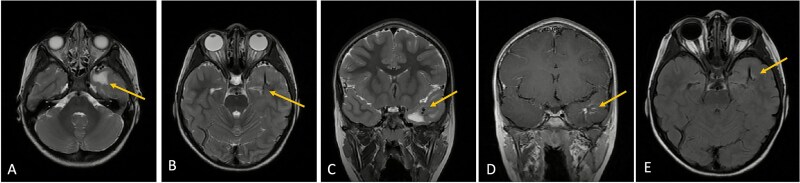
Axial lower cut (A)/axial higher cut (B)/coronal (C) T2-weighted MRI of the brain shows a prominent area of T2 hyperintensity (arrow) in the white matter of the left anterior temporal lobe, adjacent to the DVA. The DVA itself is clearly delineated on the contrasted sequence (D) and its draining veins contribute to the local anatomy (arrow). (E) Axial FLAIR (fluid-attenuated inversion recovery) highlights the abnormal hyperintensity in the left anterior temporal lobe white matter (arrow), suppressing the cerebrospinal fluid signal and making the white matter changes more conspicuous. This represents chronic changes such as gliosis or edema.

MRI with and without gadolinium contrast identified a vascular lesion consistent with a DVA in the left anterior temporal lobe. This showed typical caput medusae venous morphology. Adjacent white matter T2/FLAIR hyperintensities were present without diffusion restriction or blooming on gradient-echo sequences. No abnormal contrast enhancement was noted, and major venous sinuses were patent (Figs 1 and 2).

**Figure 3:**
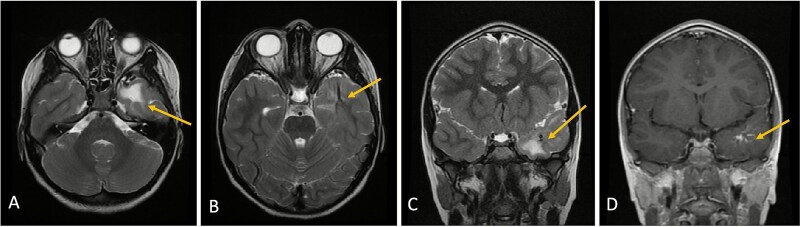
Axial lower cut (A)/axial higher cut (B)/coronal (C) T2-weighted MRI of the brain shows stable findings of a prominent area of T2 hyperintensity in the white matter of the left anterior temporal lobe, adjacent to the known DVA (arrow), which is clearly delineated on the contrasted sequence (D).

Clinical and radiological follow-up was advised. Serial MRIs at two and four months demonstrated no change in venous morphology or white matter signal. The patient’s clinical course remained uneventful, with no neurological deficits noted (Fig. 3).

## Discussion

DVAs are the most common cerebral vascular malformation, with prevalence increasing throughout childhood, likely due to maturational cerebral changes [5, 7, 8]. They form due to intrauterine arrest in venous development, leading to persistence of primitive medullary veins draining into enlarged collector veins [1, 4]. MRI findings demonstrate the pathognomonic caput medusae pattern characterized by radially converging medullary veins. Parenchymal white matter hyperintensities adjacent to DVAs represent a recognized phenomenon, likely caused by chronic venous hypertension, impaired venous drainage, or ischemia [10–13].

The pathophysiology of these white matter changes is thought to involve chronic venous stasis, which leads to increased venous pressure in the surrounding tissue. This results in blood–brain barrier disruption, venous congestion, and subsequent gliosis or edema, as evidenced by T2 and FLAIR hyperintensities on MRI. Advanced imaging studies, including diffusion-weighted and perfusion MRI, have shown altered diffusion and perfusion characteristics in the affected white matter, supporting the concept of venous hypertension and microvascular compromise. These changes are generally considered chronic and stable, reflecting long-term hemodynamic stress rather than acute injury [10–13].

Although most DVAs are asymptomatic, some pediatric cases present with seizures or headaches, and very rarely, hemorrhages occur, especially when associated with cavernous malformations [5, 14]. Our case had no such complications. Surgical intervention is generally avoided due to risk of venous infarction; conservative management with observation and serial imaging is the standard [2].

Recent Positron Emission Tomography (PET) and advanced MRI studies reveal metabolic brain alterations near DVAs, suggesting functional implications beyond structural abnormalities, which warrant further study, especially in pediatric patients [15].

The coincidental discovery of DVA in this patient following minor trauma is consistent with the asymptomatic nature of these lesions and the prevalence of neuroimaging following head injury.

## Conclusion

This case highlights an incidental DVA with white matter changes in a neurologically intact child following minor head trauma. The stable imaging and clinical findings over months support the benign nature of DVAs accompanied by chronic venous hemodynamic stress rather than acute pathology.

Recognition of white matter alterations associated with DVAs aids accurate diagnosis and guides conservative management. Further research is needed to elucidate long-term neurodevelopmental impacts in children.
